# Vulvar angiomyofibroblastoma misdiagnosed as subcutaneous lipoma: a case report and literature review

**DOI:** 10.3389/fmed.2026.1773087

**Published:** 2026-07-17

**Authors:** Di Liu, Ying Gao, Yang Chi

**Affiliations:** 1 Department of Obstetrics and Gynecology, The 964th Hospital, Changchun, Jilin, China; 2 Department of Pathology, The 964th Hospital, Changchun, Jilin, China; 3 Department of Obstetrics, The First Hospital of Jilin University, Changchun, Jilin, China

**Keywords:** angiomyofibroblastoma, immunohistochemistry, lipoma, misdiagnosis, preoperative pathological examination

## Abstract

**Background:**

Angiomyofibroblastoma (AMFB) of the vulva is a rare benign mesenchymal tumor of the vulva that affects the vulva and vagina of adult women. Often mimicked by other vulvar masses such as vestibular macrocystic glands, subcutaneous lipomas, and dermoid cysts, AMFB can be detected by histopathology. Here, we present a case where AMFB was initially misdiagnosed as a subcutaneous lipoma. By highlighting the role of preoperative pathology and imaging in the diagnostic and treatment process, we aim to improve AMFB recognition and better management.

**Case summary:**

A 59-year-old woman, postmenopausal for 10 years, reported that she first saw a vulvar mass 14 years ago, and it has gradually grown since then. A color Doppler ultrasound at another hospital detected a slightly hyperechoic mass in the fat of the left perineum measuring 7.2 × 3.0 × 4.5 cm, 36 mm from the body surface. The lesion was well circumscribed but internally heterogeneous; no intralesional blood flow was detected. No preoperative biopsy was obtained. The lesion was excised surgically. Intraoperatively, the surgeons identified a cystic mass on the lateral side of the left labium majus, measuring approximately 7.0 × 4.0 × 4.0 cm, soft, irregularly shaped, encapsulated, and clearly distinguished from adjacent tissues. Histopathological examination of the resection specimen from the left labia majora confirmed angiomyofibroblastoma. At the 3-month follow-up, no tumor recurrence was observed.

**Conclusion:**

This case confirmed that AMFB is prone to misdiagnosis. Before the operation, imaging examinations should be combined, and surgical resection should be performed after a confirmed diagnosis based on pathological and immunohistochemical examinations.

## 1 Introduction

**Figure 1:**
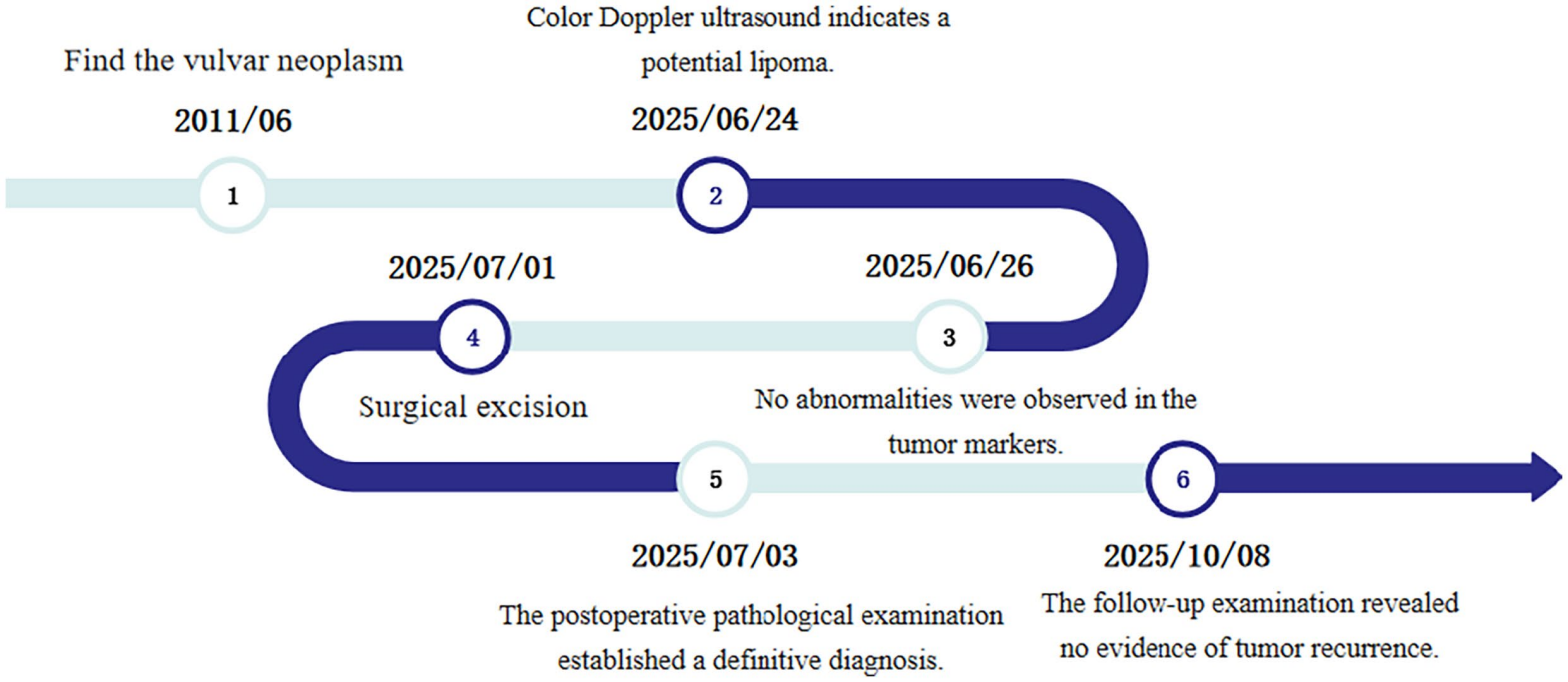
Timeline of diagnosis and treatment.

The most common vulvar tumors are hemangiomas, lipomas, fibromas, and fibroids. Angiomyofibroblastoma of the vulva was first described by Fletcher et al. It is a rare benign mesenchymal soft tissue tumor which occurs predominantly in the genital region and vagina of women of reproductive age and perimenopausal age (1). Clinically, AMFB usually develops slowly as a small painless mass. AMFB is often misdiagnosed as other vulvar lesions in practice, such as Bartholin gland cysts and lipomas (2, 3). Wolf et al. reported ultrasound usually shows a well-circumscribed hypoechoic solid mass with internal vascular signals, which is very important for imaging (4). Diagnosis is dependent on pathology, especially immunohistochemistry. We report a case of vulvar AMFB initially misdiagnosed as a lipoma in clinicoradiologically and describe the diagnostic and therapeutic course in detail (Figure 1).

## 2 Case presentation

**Figure 2:**
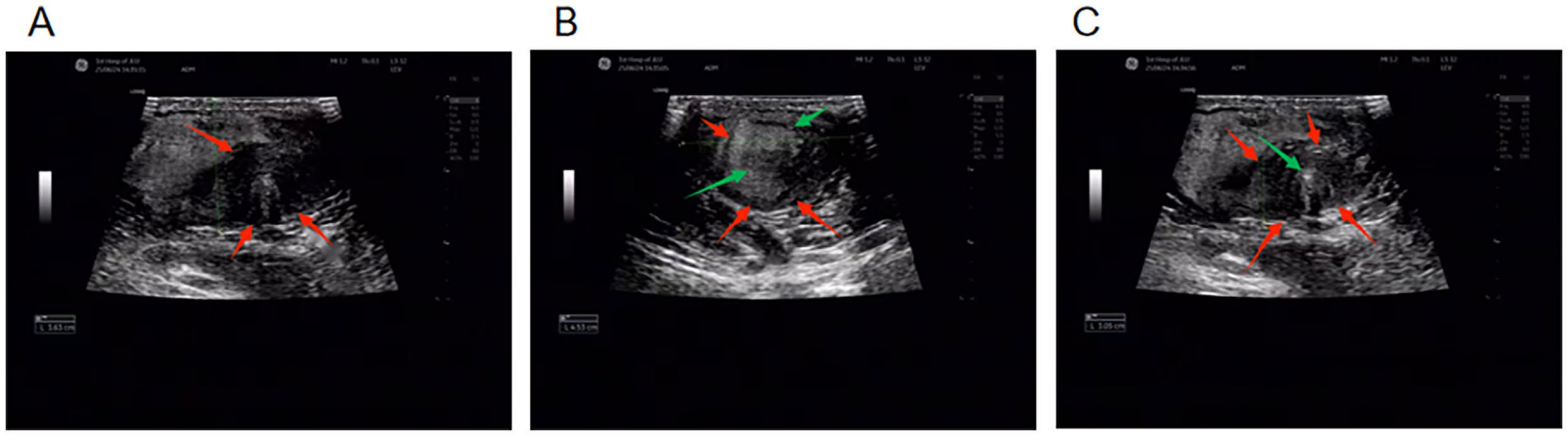
Gynecological color Doppler ultrasound examination image of the patient before admission: **(A)** Red arrow: boundary of the vulvar mass; **(B)** green arrow: slightly strong echo mass inside; **(C)** uneven echo of the mass.

A 59-year-old woman, postmenopausal for 10 years, reported that she first noticed a vulvar mass 14 years earlier and that it had grown progressively since then. At initial detection, the lesion measured approximately 2.5 × 2 × 2 cm in the left perineum and was not sampled or treated. Over the ensuing years, the mass gradually enlarged and, immediately before surgery, measured approximately 7.0 × 4.0 × 4.0 cm. A color Doppler ultrasound performed at an external institution described a slightly hyperechoic lesion within the subcutaneous fat of the left perineum, measuring 7.2 × 3.0 × 4.5 cm; the posterior margin lay 36 mm from the skin surface. The lesion had well-defined borders, a regular shape, and an internal echo pattern resembling adipose tissue but with uneven internal echoes; no significant intralesional blood flow was detected. The radiologic differential diagnosis included lipoma (Figures 2A–C). Serum tumor markers were within normal limits. Based on the clinical and imaging findings, a preoperative diagnosis of subcutaneous lipoma was made, and the patient proceeded to surgical resection after routine preoperative preparation.

**Figure 3:**
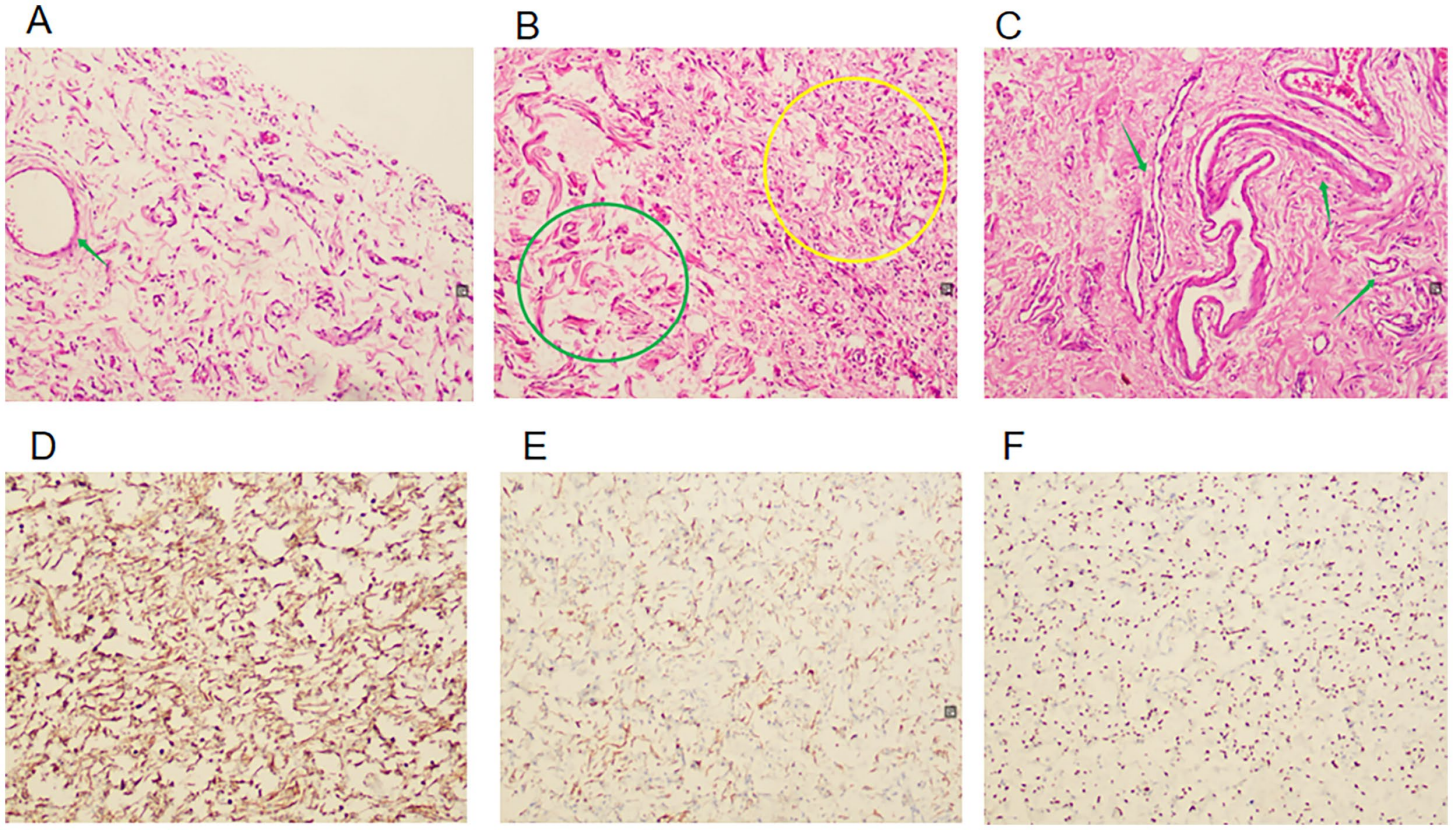
Postoperative pathological sections and specific immunohistochemical expression. **(A)** Green arrow: The tumor cells have clear boundaries and contain a large number of dilated small to medium-sized thin-walled blood vessels (HE, X10); **(B)** The tumor is alternately distributed in cell-dense and cell-loose areas, with the cell-sparse area presenting an edematous appearance (HE, X10). Green circle: Tumor cell delivery area; Yellow circle: Dense area of tumor cells; **(C)** Tumor cells grow characteristically around thin-walled blood vessels, and the interstitium is collagen-like (HE, X10); Green arrow: Distribution area of tumor cells; **(D)** Diffuse strong positive for Vimentin (IHC, X10); **(E)** Partial positive for Desmin; **(F)** Constant expression of ER.

Intraoperatively, we found a cystic mass on the left labium majus, approximately 7.0 × 4.0 × 4.0 cm. It was mostly cystic, soft, and irregularly shaped with a well-formed capsule. During the procedure, we made a longitudinal incision on the surface of the mass. After incising the skin and penetrating approximately 1.5 cm into the deep subcutaneous tissue, the capsule of the mass was visualized. The mass was completely resected along the capsule, with clearly defined surgical margins. The capsule was found to be intact during the operation. Its pedicle was superiorly and laterally on the left labium majus and extends 4 cm in the subcutaneous plane. Postoperative pathological diagnosis: Hemangioma fibroblastoma of the left major labia. Immunohistochemistry showed diffuse Vimentin-positive and focal Desmin-positive; estrogen receptor (ER) staining was positive, and progesterone receptor (PR) was positive in a small subset of cells. Smooth muscle actin (SMA) highlighted vascular structures, and S-100 was negative. The Ki-67 proliferation index was approximately 2%. CD99 was positive; caldesmon and SOX-10 were negative (Figure 3). No tumor recurred during the 3-month follow-up.

## 3 Discussion

AMFB is a rare benign mesenchymal tumor that occurs in the vulva and vaginal wall of females, with occasional cases in males (usually in the scrotum and inguinal area) (5). The cell origin of AMFB is unknown; however, evidence indicates a myofibroblastic lineage. Immunohistochemical profiles (strong vimentin expression with variable keratin and smooth muscle actin) support this interpretation (6). Some researchers also suggest that perivascular stem cells are capable of differentiating into myofibroblasts and adipocytes (7). Clinically, AMFB is usually a slow-growing mass. Microscopically, the tumor is alternating dense and poor zones, and the hypocellular areas are often mucoid or edematous (2). Tumor cells cluster around thin-walled blood vessels, and the stroma contains variable amounts of collagen (2, 8). Neoplastic cells express vimentin and desmin, partially express SMA and MSA, ER- and PR-positive cells; CD34 and S-100 are negative cells, and desmin expression may decrease after menopause, and the Ki-67 proliferation index is low (9). Recent studies show robust CYP2E1 immunoreactivity in AMFB (10).

Clinically, AMFB lesions most commonly range from 0.5 to 5 cm in diameter, although occasional tumors can reach 10 cm. Their clinical appearance and size frequently lead to misdiagnosis as Bartholin gland cysts, subcutaneous lipomas, inguinal hernias, or other similar lesions (11).

Pathologically and immunohistochemically, this tumor is most difficult to distinguish from three entities: (1) Aggressive angiomyxoma (AAM): AAM is characterized by rapid progression and brisk tumor growth, with lesions often reaching 5 centimeters in diameter and frequently infiltrating adjacent tissues, which complicates complete surgical excision; nevertheless, it typically lacks metastatic behavior. By contrast, AMFB is usually smaller, with a tumor size of less than 5 cm in most cases (12). Under microscopic examination, the cells were uniformly distributed rather than arranged in alternating architectural zones and showed infiltrative margins, often alongside large, thick-walled blood vessels. Immunohistochemistry demonstrated that tumor cells in both AAM and AMFB consistently expressed vimentin, estrogen receptor, and progesterone receptor, while S-100 protein was negative. In contrast, desmin and *α*-SMA expression varied among individual cases (13, 14). (2) Cellular angiofibroma (CA): Spindle-shaped cells, prominent vascular channels, mature adipose tissue; immunohistochemistry often shows focal positivity for ER, PR, CD34, SMA, and desmin; FISH frequently shows the deletion of RB1 at 13q14. (3) Superficial angiomyxoma (SA): reticular dermis with poorly defined margins, focal lobular or multinodular proliferation; loose spindle or stellate fibroblasts lack nuclear atypia; CD34 commonly shows on immunohistochemistry; SMA or desmin may be focally positive in some cases; ER, PR, and S-100 protein are consistently negative (6).

This case presents a vulvar mass that has remained for 14 years. Although slowly expanding, the disease remains poorly defined, and the patient is asymptomatic. After surgical removal, the tumor is smooth enough to be surgically removed. Immunohistochemistry of the resected specimen shows diffuse strong vimentin staining and focal desmin positivity in tumor cells. The main problem in this case was that the lesion was mistakenly diagnosed as a lipoma based only on the gynecological color Doppler ultrasound, and no tumor core needle biopsy was obtained. Although the intraoperative appearance differed from that of a typical subcutaneous lipoma, no frozen sections were obtained to provide rapid pathological confirmation. Postoperative pathology showed angiomyofibroblastoma (AMFB). If the tumor had been malignant, the surgical margins would not have been adequate, and a second operation would have been required, and subsequent management would have been difficult. The postoperative follow-up period is also relatively short. If AMFB resection is incomplete during surgery, AMFB may recur after a longer interval. Therefore, continuous monitoring should be maintained thereafter.

Some reports suggest that some patients with AMFB may develop malignant sarcomatoid transformation after tumor resection (15). In conclusion, once AMFB has been diagnosed, complete surgical resection is advised for less risk of recurrence and malignant development, and close postoperative monitoring is important. In the present case report, complete surgical resection was done, and the patient was monitored for 3 months postoperatively. No tumor recurrence or metastases were detected during that period; follow-up is ongoing for long-term results.

AMFB is a rare benign vulvar tumor that is often misdiagnosed because of overlap with other lesions. Preoperative imaging should be performed frequently to characterize the extent of the lesion, and definitive diagnosis should be based on histopathological and immunohistochemical evaluation.

## Data Availability

The original contributions presented in the study are included in the article/supplementary material, further inquiries can be directed to the corresponding author/s.
